# The Tub in Obstetrics

**Published:** 1901-06-22

**Authors:** 


### THE TUB IN OBSTETRICS.

As an aid to cleanliness the tub is greatly to be recommended. Still even the tub has its drawbacks, and certain scientifically minded foreigners have lately been inquiring whether in some circumstances it may not do harm rather than good. The particular experiments in question were carried out with the object of ascertaining how far it was desirable that women who were about to be confined should be given the complete bath which in some institutions is regarded as the routine and proper antecedent of the act of parturition, and are recorded in the *Centralblatt f. Gynäkologie* by Dr. Sticher and Dr. Stroganoff. The first of these gentlemen after adding to the water of the bath pure cultures of the bacillus prodigiosus, a microbe which does not occur in the normal vagina, was able to recover the bacillus after the bath from the vaginas of both primiparæ and multiparæ, while Dr. Stroganoff by making use of the chemical reaction of starch and iodide of potassium was able to demonstrate that the water of the bath gained access to the interior of the vagina. Hence they deduce on the one hand the necessity of disinfecting the bath itself between the successive occasions of its use, and on the other the questionable propriety of using the ordinary tub bath on such occasions at all, seeing that the skin of most people is more or less coated with dirt which contains microbes in abundance, some of them pathogenic. Dr. Stroganoff recommends that in place of the tub bath some form of Russian or needle bath should be employed. Where it can be obtained there can be no doubt of the great superiority of the needle bath over the tub bath as a means to cleanliness. We have again and again advised that arrangements on this principle should be placed in our public baths (they are in some places) instead of the stupid old-fashioned tub bath, which is, perhaps, the most ineffectual cleansing device which could be invented. When a dirty person takes a tub the dirt combines with the soap and floats up as a scum upon the water. This deposits itself all over the skin on emerging from the bath, and is partly rubbed in again, and partly wiped on to the towel; whereas with a quarter the quantity of water in the form of a needle spray it all goes down the drain, and the man emerges clean. A tub is all right for those who tub regularly, but an occasional tub is but a distribution of dirt. Some goes back to the skin, some sticks to the sides of the bath for the delectation of the next comer, most goes on to the towel, while a little goes down the drain! All of which is just the opposite of what ought to happen.

